# *P2RX7* gene variation mediates the effect of childhood adversity and recent stress on the severity of depressive symptoms

**DOI:** 10.1371/journal.pone.0252766

**Published:** 2021-06-10

**Authors:** Zsuliet Kristof, Nora Eszlari, Sara Sutori, Zsofia Gal, Dora Torok, Daniel Baksa, Peter Petschner, Beata Sperlagh, Ian M. Anderson, John Francis William Deakin, Gabriella Juhasz, Gyorgy Bagdy, Xenia Gonda

**Affiliations:** 1 Doctoral School of Mental Health Sciences, Semmelweis University, Budapest, Hungary; 2 Laboratory of Molecular Pharmacology, Institute of Experimental Medicine, Budapest, Hungary; 3 Department of Psychiatry and Psychotherapy, Semmelweis University, Budapest, Hungary; 4 Faculty of Pharmacy, Department of Pharmacodynamics, Semmelweis University, Budapest, Hungary; 5 NAP-2-SE New Antidepressant Target Research Group, Hungarian Brain Research Program, Semmelweis University, Budapest, Hungary; 6 Faculty of Humanities and Social Sciences, Institute of Psychology, Pazmany Peter Catholic University, Budapest, Hungary; 7 SE-NAP-2 Genetic Brain Imaging Migraine Research Group, Hungarian Brain Research Program, Semmelweis University, Budapest, Hungary; 8 MTA-SE Neuropsychopharmacology and Neurochemistry Research Group, Hungarian Academy of Sciences, Semmelweis University, Budapest, Hungary; 9 Faculty of Biological, Division of Neuroscience and Experimental Psychology, Neuroscience and Psychiatry Unit, School of Biological Sciences, Medical and Human Sciences, The University of Manchester and Manchester Academic Health Sciences Centre, Manchester, United Kingdom; Universita degli Studi Europea di Roma, ITALY

## Abstract

The P2X purinoceptor 7 (P2RX7) mediates inflammatory microglial responses and is implicated in neuroimmune mechanisms of depression and neurodegenerative disorders. A number of studies suggest that psychosocial stress may precipitate depression through immune activation. Genetic association studies of *P2RX7* variants with depression have been inconclusive. However, nearly all studies have focused on only one single-nucleotide polymorphism (SNP) and have not considered interaction with psychosocial stress. We investigated the effect of several variations in *P2RX7* gene using a clumping method in interaction with early adversities and recent stress on depression severity. 1752 subjects provided information on childhood adversities, recent life events, and current depression severity. Participants were genotyped for 681 SNPs in the *P2RX7* gene, 335 of them passed quality control and were entered into linear regression models followed by a clumping procedure for main effect and interactions. No significant main effect was observed. Rs74892325 emerged as a top SNP for interaction with childhood adversities and rs61953400 for interaction with recent life events. Our study is the first to investigate several variants in the *P2RX7* gene and in interaction with two types of stress, extending our understanding of neuroinflammation in depression, and supporting that the majority of genes influence depression by enhancing sensitivity to stressors.

## Introduction

Depression has a high economic and social impact and leads to significant suffering, while 35% of patients are resistant to currently available treatments [1]. There is a clear need for better understanding of the neurobiology of depression, and more effective and novel diagnostics, treatment targets, and action mechanisms. Most antidepressants act on monoamine systems with a slow onset of action and partial response and remission is common. Furthermore, we still lack biological markers for underlying neural mechanisms of depression that can identify subtypes and personalise treatment. Recent years have seen a major shift in emphasis from a neuron-centric view of depression towards a recognition that neurons and glia act in partnership in mediating brain homeostasis and that abnormal neuron-glial interaction is central to the development of mood pathology [2–4].

Glial cells, and especially microglia have a crucial role in maintaining the immune health of the brain. They sense foreign proteins and signals of neuronal stress and respond by generating proinflammatory factors including TNF-α, IL-6 and IL-1β that amplify the protective immune response. In morbid states such as autoimmunity, neuroinflammation leads to neuronal dysfunction and damage. Increasing evidence suggests that dysfunctional neuroimmune activation plays a causal role in the onset and maintenance of depressive illness [2,5–8]. Most directly, several studies report increased in-vivo radioligand binding to the translocator protein, a marker of activated microglia, in depression [9,10]. Furthermore, meta-analyses suggest anti-inflammatory drugs, notably the antimicroglia inhibitor antibiotic minocycline [11], have antidepressant efficacy.

P2X purinoceptor 7 (P2RX7), showing abundant expression in microglia [6], is a cell surface sensor of danger signal activated by a sharp increase in extracellular ATP, a damage-associated molecular pattern (DAMP) indicative of neuronal damage. Hallmark downstream effects of P2RX7 activation include NLRP3 inflammasome complex mediated proinflammatory IL-1β and IL-18 release [2,6,12,13]. Stress is a major environmental etiological factor in mood disorders [14–16], and P2RX7 activation has been associated with an inflammatory and stress-mediated depression-like phenotype [2,17,18] with enhanced IL-1β release contributing to stress-induced depression [2]. The potential role of P2RX7 in depression is also reflected by the fact that its activity and hippocampal expression is selectively modulated by different antidepressants and by stress exposure [19].

Variation in the *P2RX7* gene, encoding the P2X7 receptor, located on 12q24.31, has been implicated in both bipolar disorder (BD) and major depressive disorder (MDD) [20], but the majority of studies so far focused on a single SNP, rs2230912 [21–23], where the minor G allele is associated with a gain-of-function phenotype for increased IL-1β release in monocytes [24] and possibly also in microglia, leading to neuroinflammation and increased risk for mood disorders [23,25]. Furthermore, although the effects of the P2X7 receptor are manifested in transducing danger signals and regulating neurochemistry under stress [19] and it has been hypothesised that *P2RX7* predisposes to depression in interaction with life stress [26], studies investigating the role of variation in *P2RX7* in depression have not considered the effects of stress.

Thus, while rapidly emerging knowledge points to the regulatory role of purinergic signalling in mood-related behaviour, stress reactivity, and their pathological alterations, with novel brain-penetrant *P2RX7* antagonists (JNJ-54175446, JNJ-55308942) in phase 2 and 3 clinical trials for the treatment of depression [2,26], we still lack proper understanding of the role of variation in *P2RX7* in the background of depressive-like behaviours. Given our increasing understanding that the majority of genes implicated as etiological factors in the development of depression have a role in mediating response to stressors rather than directly influencing risk, *P2RX7* may be an especially relevant target [27,28]. Furthermore, a more sophisticated insight on the role of stressors and gene x environment interactions is needed as life events may play both a predisposing role, leading to the development of a diatheses, in case of early, distal exposures, and a trigger role in case of recent, proximal exposures. Therefore, in our study, we focused on the effects of variation in the *P2RX7* gene in interaction with early childhood adversities and recent life events on current depressive symptoms in a large general European sample.

## Materials and methods

### Study population

This study was part of the NewMood study (New Molecules in Mood Disorders, Sixth Framework Program of the EU, LSHM-CT-2004-503474) and was funded by the European Union. Participants aged between 18–60 years were recruited from the general population through advertisements, a website, and general practices in greater Manchester and Budapest. 1752 non-related subjects of European white ethnic origin (501 males, 1251 females) provided self-reported data on gender, age, recent stress, childhood adversities, current depression among other variables by filling out a questionnaire pack, and provided genetic data by a saliva sampling kit. As NewMood focused on a general population with a continuum approach to affective symptoms and disorders, our sample included previously depressed, currently depressed, and never depressed participants as well. In the present study only information on current depressive symptom severity was included. More details about the population sample can be found in our previously published reports [29–31]. All participants provided written informed consent. The study was carried out in accordance with the Declaration of Helsinki, and it was approved by the Scientific and Research Ethics Committee of the Medical Research Council, Budapest, Hungary (ad.225/KO/2005.; ad.323-60/2005-1018EKU), and by the North Manchester Local Research Ethics Committee, Manchester, United Kingdom (REC reference number 05/Q1406/26).

### Phenotypes

We applied a continuum approach in a general population sample to capture current severity of depressive symptoms, rather than ascertaining clinical levels of depression and employing a case-control design. The Brief Symptom Inventory (BSI) was used to measure current levels of depression [32]. Each item was scored between 0–4 depending on the distress caused. Depression score (BSI-depression) was calculated as the sum of depression and additional item scores divided by the number of completed items. The use of BSI for capturing current depressive symptoms has been validated in a previous study using a subsample of our study population with the Montgomery-Asberg Depression Rating Scale (MADRS) administered by trained interviewers [33].

In our study we measured two types of stress, early childhood adversity (CHA) and recent stressful life events (RLE). The childhood adversity measure was derived from the Childhood Trauma Questionnaire (CTQ) [34], including four items about emotional and physical abuse and emotional and physical neglect, and two items about the loss of parents. We had validated this short childhood adversity measure with the 28-item CTQ within a subpopulation of our sample, obtaining a high correlation between the original and derived measures [33]. The sum of item scores was used in the analyses. Recent stressful life events (RLE) occurring in the previous year related to financial difficulties, illnesses/injuries, personal problems, and intimate relationship or social network difficulties were ascertained by the List of Threatening Experiences [35,36]. The number of recent negative life events (RLEs) was used in the statistical analyses.

Following our analyses, solely for illustration purposes, RLE and CHA scores were both grouped into three categories to reflect severity of exposure (RLE: low = 0, medium = 1, high = 2 or more; and CHA: low = 0–3, medium = 4–6, high = 7 or more) based on our previous studies [33,37].

### Genotyping

Participants provided buccal mucosa cells collected by a cytology brush (Cytobrush plus C0012, Durbin PLC). Genomic DNA was extracted according to the protocol of Freeman et al. [38]. Genotyping was performed by Illumina’s CoreExom PsychChip. All laboratory work was performed under the ISO 9001:2000 quality management requirements and was blinded with regard to phenotype.

### Statistical analyses

We analysed all SNPs in the region of *P2RX7* available in the NewMood database and surviving quality control steps using linear regression models, followed by a clumping procedure based on linkage disequilibrium (LD) estimates between the SNPs. Results of top SNPs from representing correlated SNPs in individual clumps are reported.

**Fig 1:**
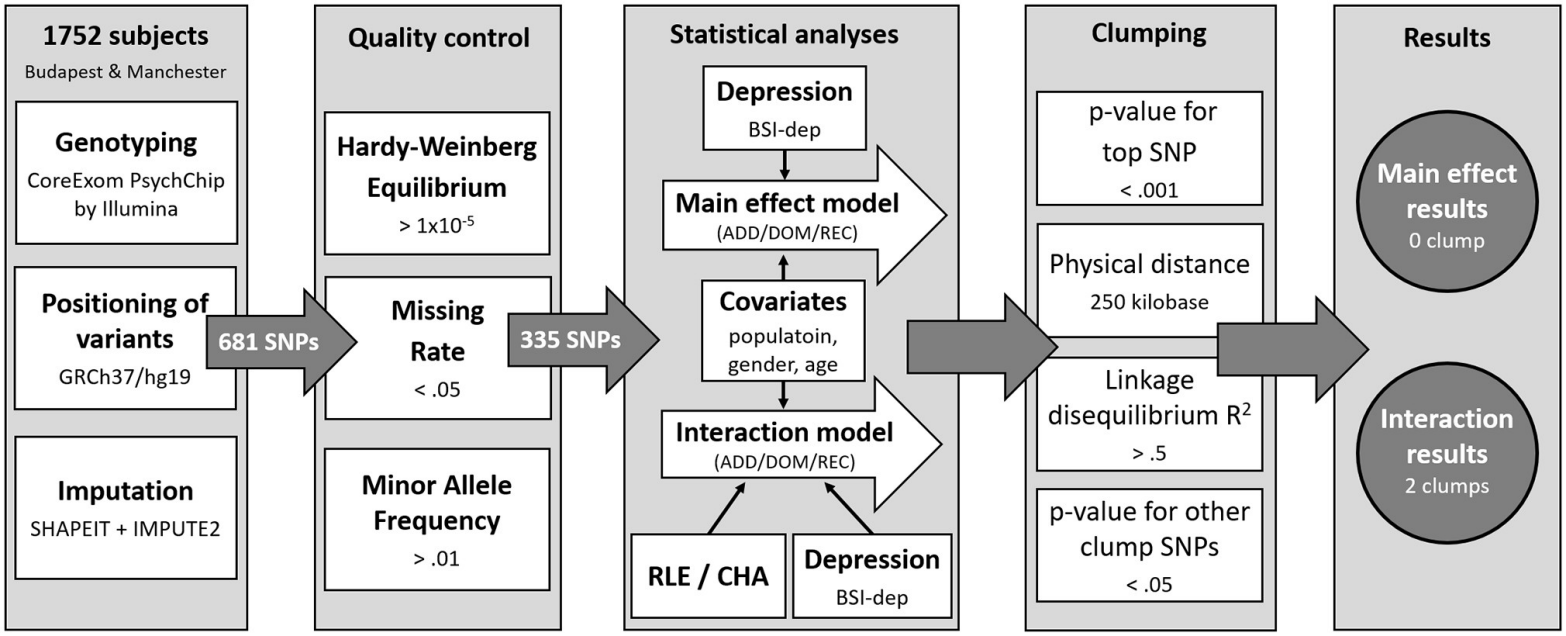
Methods of investigating the effects of variation in *P2RX7* in interaction with childhood adversities and recent life events on current depression: Study population, quality control steps and statistical analyses. RLE: recent life events; CHA: Childhood adversities; BSI-Dep–Brief Symptom Inventory depression score; ADD: additive model; DOM: dominant model; REC: recessive model.

IBM SPSS Statistics 25 was used to calculate descriptive statistics and to run univariate general linear models solely for visualization purposes. Plink v1.90 was used to calculate missingness rate (MR), Hardy-Weinberg equilibrium (HWE) and minor allele frequency (MAF) as part of quality control steps, for clumping, and to build linear regression models on BSI-depression. Besides testing the main effects of genetic variants on BSI-depression, interaction models to test for gene x environment interactions with RLE and CHA were also run (Fig 1). Analyses were supported by scripts individually written in R 3.0.2 (R Core Team, 2013).

First, we created (.map and.ped) files that contained only those SNPs which fulfilled the criteria of the quality control and only analysed those SNPs that survived this procedure. Genotyping provided a dataset incorporating 681 SNPs. Quality control entailed the calculation of Hardy-Weinberg Equilibrium (HWE; >1x10^-5^), missing rates (MR; < .05), and minor allele frequencies (MAF; >.01). After quality control, 335 SNPs remained to be entered into statistical analysis. In our Plink linear regression models population, sex and age were covariates in all analyses. When testing an SNP × CHA/RLE interaction effect, main effects of both the SNP and CHA/RLE were included as covariates in the model.

After running the regressions, SNPs were clumped both for main effect and for interactions using the CLUMP function in Plink. The four parameters used for clumping were as follows: maximum p-value of the clump’s top SNP was set at 0.001; physical distance with it was 250 kilobase; minimum linkage disequilibrium R^2^ with it was 0.5; and maximum p-value for the clump’s other SNPs was 0.05. Data on all *P2RX7* SNPs in the original NewMood database, including quality control and regression results are shown in S1 and S2 Tables.

All analyses were run according to additive, dominant and recessive models. Nominal significance threshold was p<0.05. To correct for multiple comparisons in analyses for each of the above outcome variables, Benjamini-Hochberg false discovery rate (FDR) Q-values were calculated. Results with a Q-value of ≤0.05 were considered as significant.

The data presented in this study are openly available in FigShare at 10.6084/m9.figshare.13483011 [39].

## Results

### Descriptive statistics

Descriptives of our study sample are provided in Table 1.

**Table 1:** Descriptive statistics of the study sample.

Gender	n	%		
Male	501	28.6%		
Female	1251	71.4%		
	Minimum	Maximum	Mean	SEM
Age	18	60	32.56	0.25
Depression score	0	4	0.84	0.02
Childhood adversity	0	16	3.28	0.08
Recent negative life events	0	8	1.21	0.03

Depressive symptom scores were measured by the Brief Symptom Inventory (BSI). SEM: standard error of mean.

### Effects of variation in *P2RX7* on current depressive symptoms: Main effects and interaction with childhood adversities (CHA) and recent life events (RLE)

Our statistical analyses did not identify any SNPs with a nominally significant main effect on current depression (BSI-depression) thus clumps could not be identified. Our analyses for interaction either with childhood adversities (CHA) or recent life events (RLE) yielded one clump in each case. For interaction with CHA, the clump contained 10 SNPs, with rs74892325 as the top SNP, while for interaction with RLE the clump contained 2 SNPs with rs61953400 as the most significant SNP (Table 2).

**Table 2:** Significant clumps of *P2RX7* SNPs interacting with early childhood adversities (CHA) and recent life events (RLE) on current depression symptoms.

*P2RX7* x CHA						
	*ß*	*95% C*.*I*.	*p*	*Hardy*	*MAF*	*Missing*
**rs74892325**	-0.072	-0.108 − -0.036	<0.0001	0.830	0.058	0.010
rs2686372	-0.073	-0.110 − -0.036	0.0001	1	0.055	0.012
rs112265934	-0.072	-0.109 − -0.035	0.0001	1	0.054	0.008
rs113760750	-0.071	-0.108 − -0.034	0.0002	1	0.055	0.007
rs75009692	-0.073	-0.111 − -0.035	0.0002	0.813	0.052	0.014
rs77594915	-0.072	-0.110 − -0.035	0.0002	1	0.054	0.010
rs113810203	-0.072	-0.109 − -0.034	0.0002	1	0.054	0.010
rs3751145	-0.071	-0.109 − -0.034	0.0002	1	0.054	0.010
rs112956506	-0.071	-0.109 − -0.034	0.0002	1	0.054	0.009
rs112803307	-0.072	-0.117 − -0.026	0.0020	0.466	0.035	0.021
*P2X7* x RLE						
	*ß*	*95% C*.*I*.	*p*	*Hardy*	*MAF*	*Missing*
**rs61953400**	0.209	0.087–0.331	0.0008	0.582	0.022	0.009
rs61953398	0.182	0.070–0.293	0.0014	0.720	0.033	0.012

CHA: Childhood adversities; RLE: Recent life events. Bold type denotes top SNPs in each clump.

Both top *P2X7R* SNPs were in Hardy–Weinberg equilibrium in the sample (rs74892325: p = 0.8295, rs61953400: p = 0.5823). For rs74892325, T is the minor allele, with an allele frequency of 0.05771. For rs61953400, A is the minor allele, yielding an allele frequency of 0.0219.

Both top SNPs were tested in main effect models as well as for interaction with both CHA and RLE (Table 3). None of the top SNPs had a significant main effect on BSI-depression.

**Table 3:** Main effects and interactions with childhood adversities (CHA) and recent life events (RLE) of *P2RX7* tops SNPs rs74892325 and rs61953400 on current depression symptom severity.

		ADD				DOM				REC			
		*ß*	*95% C*.*I*.	*p*	*FDR Q*	*ß*	*95% C*.*I*.	*p value*	*FDR Q*	*ß*	*95% C*.*I*.	*p*	*FDR Q*
*rs74892325*	Main effect	-0.102	-0.227–0.023	0.1101	0.2202	-0.114	-0.244–0.016	0.0861	0.1968	0.130	-0.638–0.898	0.7399	0.7892
	CHA interaction	-0.070	-0.104 - -0.035	**<0.0001**	**0.0006**	-0.072	-0.108 - -0.036	**<0.0001**	**0.0006**	-0.070	-0.311–0.172	0.5713	0.7031
	RLE interaction	-0.048	-0.143–0.048	0.3263	0.4351	-0.053	-0.151–0.044	0.2843	0.4135	0.048	-0.948–1.044	0.9241	0.9241
*rs61953400*	Main effect	0.126	-0.073–0.326	0.2139	0.3422	0.138	-0.066–0.341	0.1849	0.3287	-0.429	-2.151–1.293	0.6253	0.7146
	CHA interaction	0.045	-0.005–0.096	0.0752	0.1968	0.045	-0.005–0.095	0.0795	0.1968				
	RLE interaction	0.194	0.074–0.314	**0.0015**	**0.0062**	0.209	0.087–0.331	**0.0008**	**0.0043**				

Depressive symptom scores were measured by Brief Symptom Inventory (BSI). ADD, DOM, REC: additive, dominant and recessive heritability models. CHA: Childhood Adversity; FDR: false discovery rate; RLE: recent life stress. Bold type denotes significant P-values surviving correction for multiple testing (P<0.05, FDR Q<0.05).

**Fig 2:**
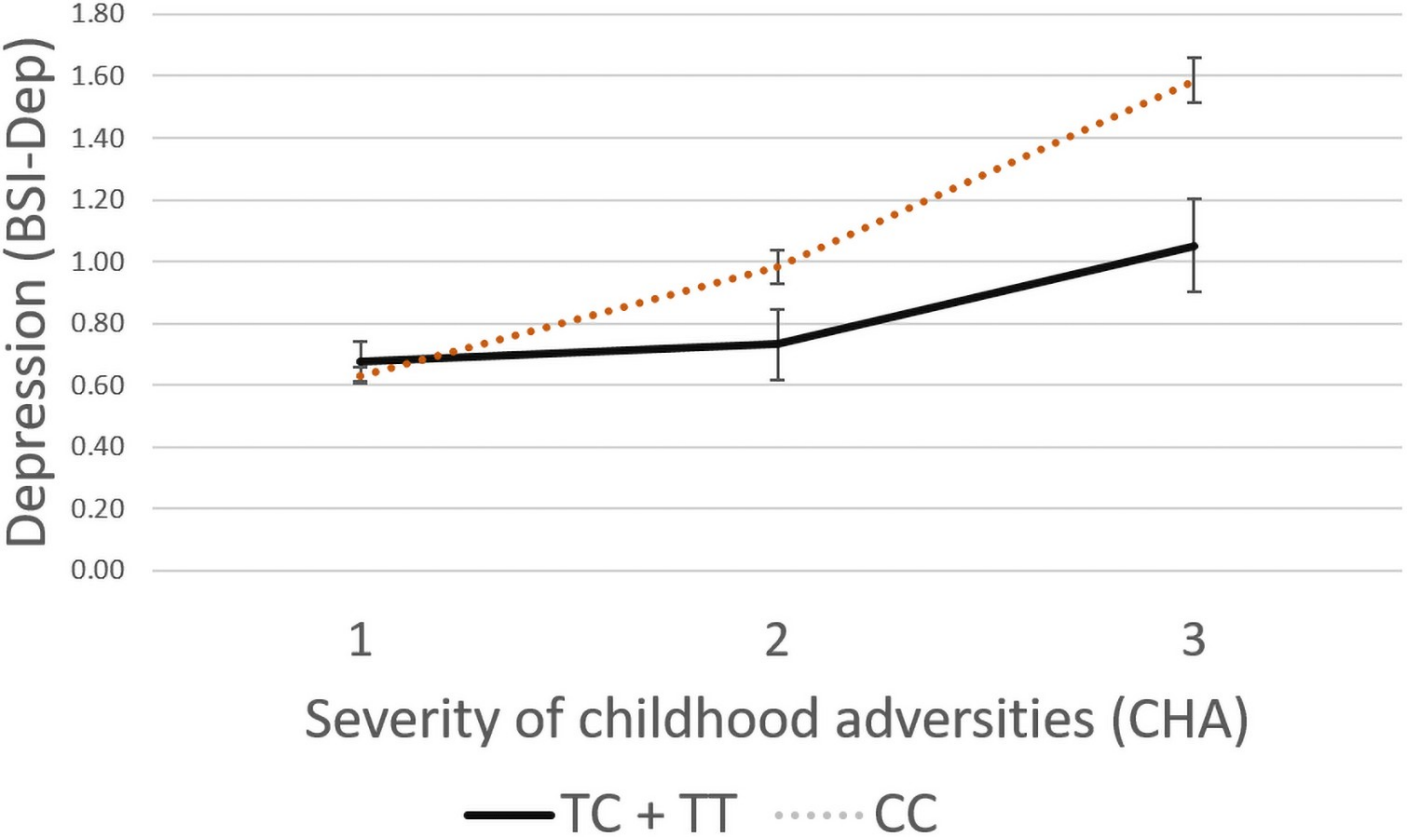
Linear regression analyses indicated a significant interaction between *P2RX7* rs74892325 and exposure to childhood adversities on current depression, with the minor T allele as a protective allele. Linear regression indicated a significant interaction between *P2RX7* rs74892325 genotype and severe childhood adversities (CHA) on current depression scores (BSI-DEP) according to the additive (p<0.0001, FDR Q = 0.0006) and dominant (p<0.0001, FDR Q = 0.0006) models. Presence of the minor T allele was associated with lower depression scores in subjects exposed to severe recent life events conveying a protective effect. On the vertical axis weighted depression (BSI-Dep) scores are shown. The horizontal axis shows severity of childhood adversities (CHA) categorised as 1: low = 0–3, 2: medium = 4–6, 3: high = 7 or more. While regression models were used in the analyses, the figure depicts results from ANOVA solely for visualisation purposes. Mean and SEM values are displayed.

In case of rs74892325, no significant interaction effect emerged with recent life events (ADD: p = 0.3263, DOM: p = 0.2843, REC: p = 0.9241), but a significant interaction with childhood adversity on current depressive symptoms was found in both additive (p<0.0001) and dominant (p<0.0001) but not in recessive (p = 0.5713) models which remained significant after correction for multiple testing (FDR Q = 0.0007 and FDR Q = 0.0007 for additive and dominant models respectively). Rs74892325 minor T allele carriers scored significantly lower on BSI-depression scale if they were exposed to moderate or severe childhood maltreatment reflecting a protective effect for the minor allele (Fig 2).

**Fig 3:**
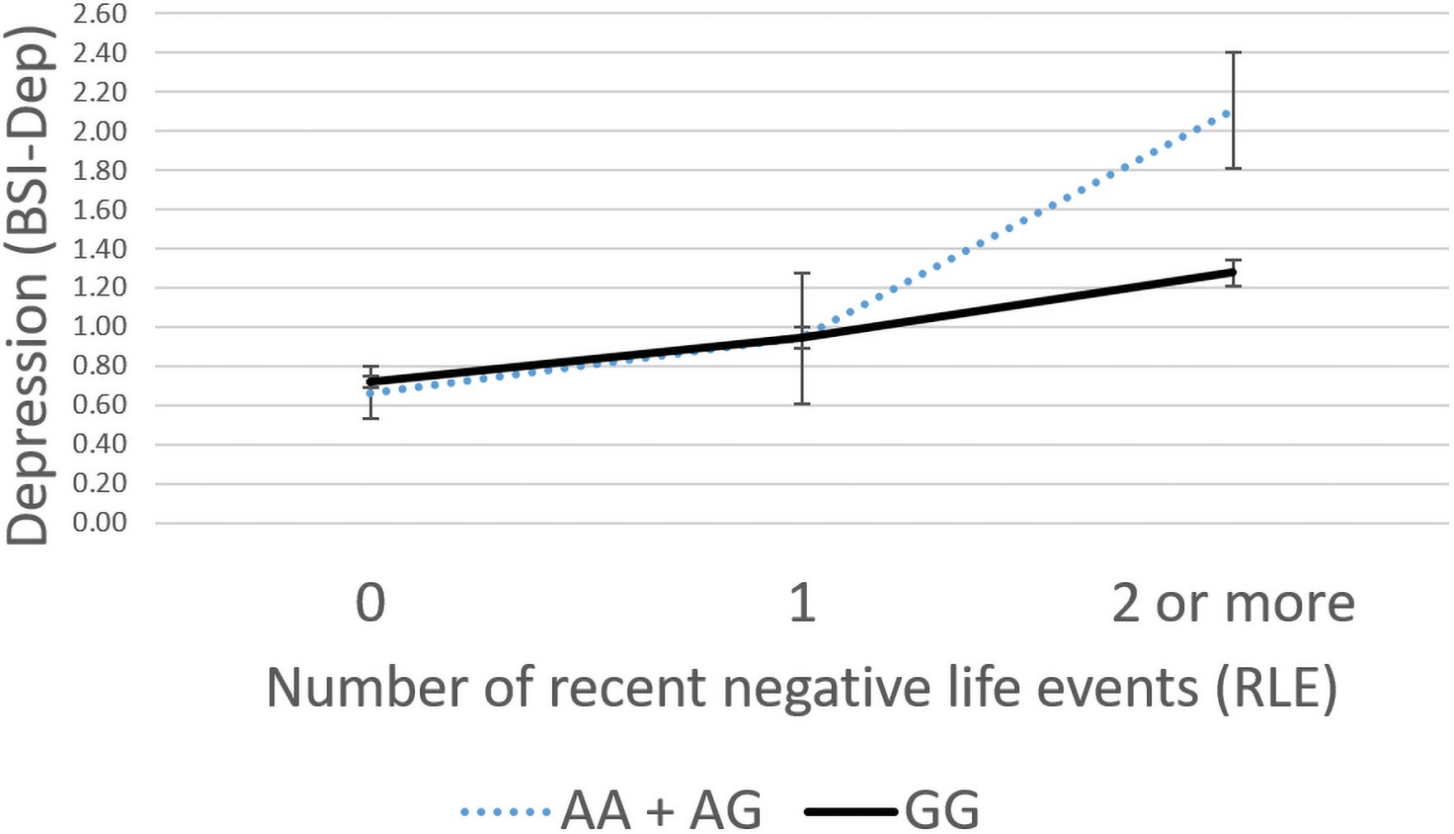
Linear regression analyses indicated a significant interaction between *P2RX7* rs61953400 and exposure to recent life events on current depression, with the minor A allele as a risk allele. Linear regression indicated a significant interaction between *P2RX7* rs61953400 genotype and recent stressful life events (RLE) on current depression scores (BSI-DEP) according to the additive (p<0.0015, FDR Q = 0.0062) and dominant (p<0.0001, FDR Q = 0.0043) models. Presence of the minor A allele was associated with higher depression scores in subjects exposed to severe recent life events conveying a risk effect. On the vertical axis weighted depression (BSI-Dep) scores are shown. The horizontal axis shows recent life events (RLE) occurring within the past year as measured by the List of Threatening Experiences [36] categorised as 0 –low exposure (0 events reported) 1 –medium exposure (1 event reported) or 3 –high exposure (2 or more events reported). While regression models were used in the analyses, the figure depicts results from ANOVA solely for visualisation purposes. Mean and SEM values are displayed.

In case of rs61953400, no significant interaction effect with childhood adversities was observed (ADD: p = 0.0752, DOM: p = 0.0795), but a nominally significant interaction with recent life events was found in both additive (p = 0.0015) and dominant (p = 0.0008) models which remained significant after correction for multiple testing (FDR Q = 0.0062 and FDR Q = 0.0043 for additive and dominant models, respectively) (Table 3). Presence of the minor A allele was associated with increased BSI-depression scores in those exposed to severe recent life events (RLE) reflecting a risk effect (Fig 3). Because the particularly limited number of cases of homozygous carriers of the minor allele (A) in certain exposure groups, the recessive models could not be calculated and the results are not shown.

### In silico characterisation and functional prediction of identified top SNPs *rs74892325* and *rs61953400*

**Fig 4:**
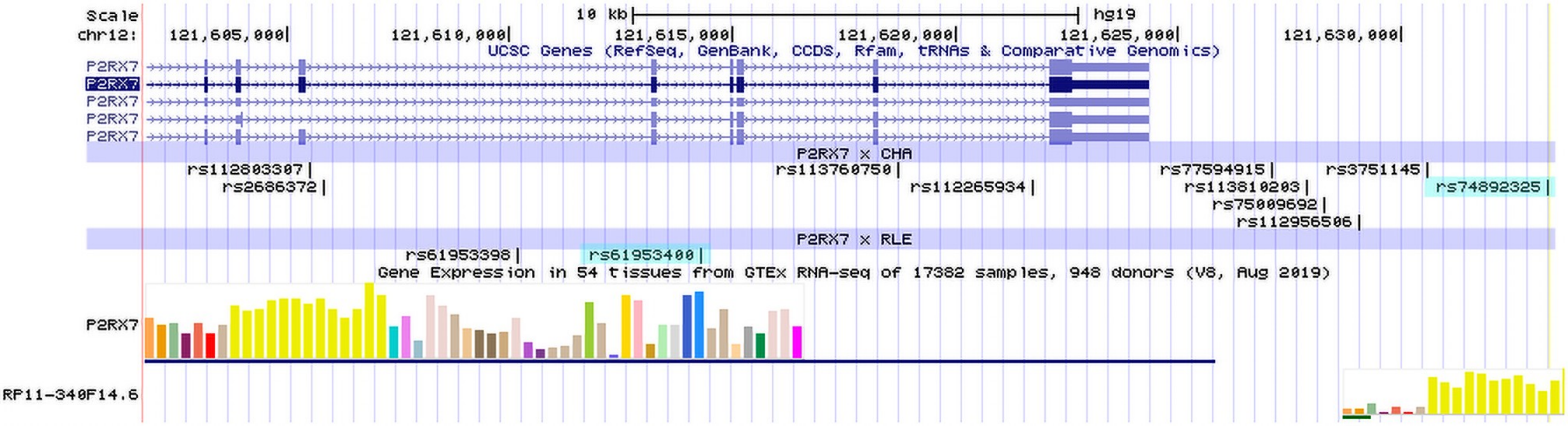
Genomic location of significant SNPs identified in the clumping procedure. Significant SNPs identified in the analysis with an effect on current depression symptoms in interaction with childhood adversities are denoted as *P2RX7* x CHA; significant SNPs identified in the analysis with an effect on current depression symptoms in interaction with recent life event are denoted as *P2RX7* x RLE; top SNPs in the two analyses are marked with light blue. In the gene expression part of the image, yellow bars indicate expression in nervous tissues. UCSC Genome Browser on Human Feb. 2009 (GRCh37/hg19) was used to visualize the location of polymorphisms on *P2RX7* gene.

Genomic location of significant SNPs identified in the clumping procedure are shown in Fig 4. Search in LitVar (https://www.ncbi.nlm.nih.gov/CBBresearch/Lu/Demo/LitVar/#!?query=), dbSNP (https://www.ncbi.nlm.nih.gov/snp/), and GWAS catalog database (https://www.ebi.ac.uk/gwas/) did not identify any relevant information regarding the two SNPs or any further SNPs in our clumps. FuncPred (www.funcpred.com) tool could also not identify any known functional effect of our top SNPs except for rs2686372 intronic variant (in the CHA interaction clump), where in function prediction we observed a 0.435 conservation score.

## Discussion

**Fig 5:**
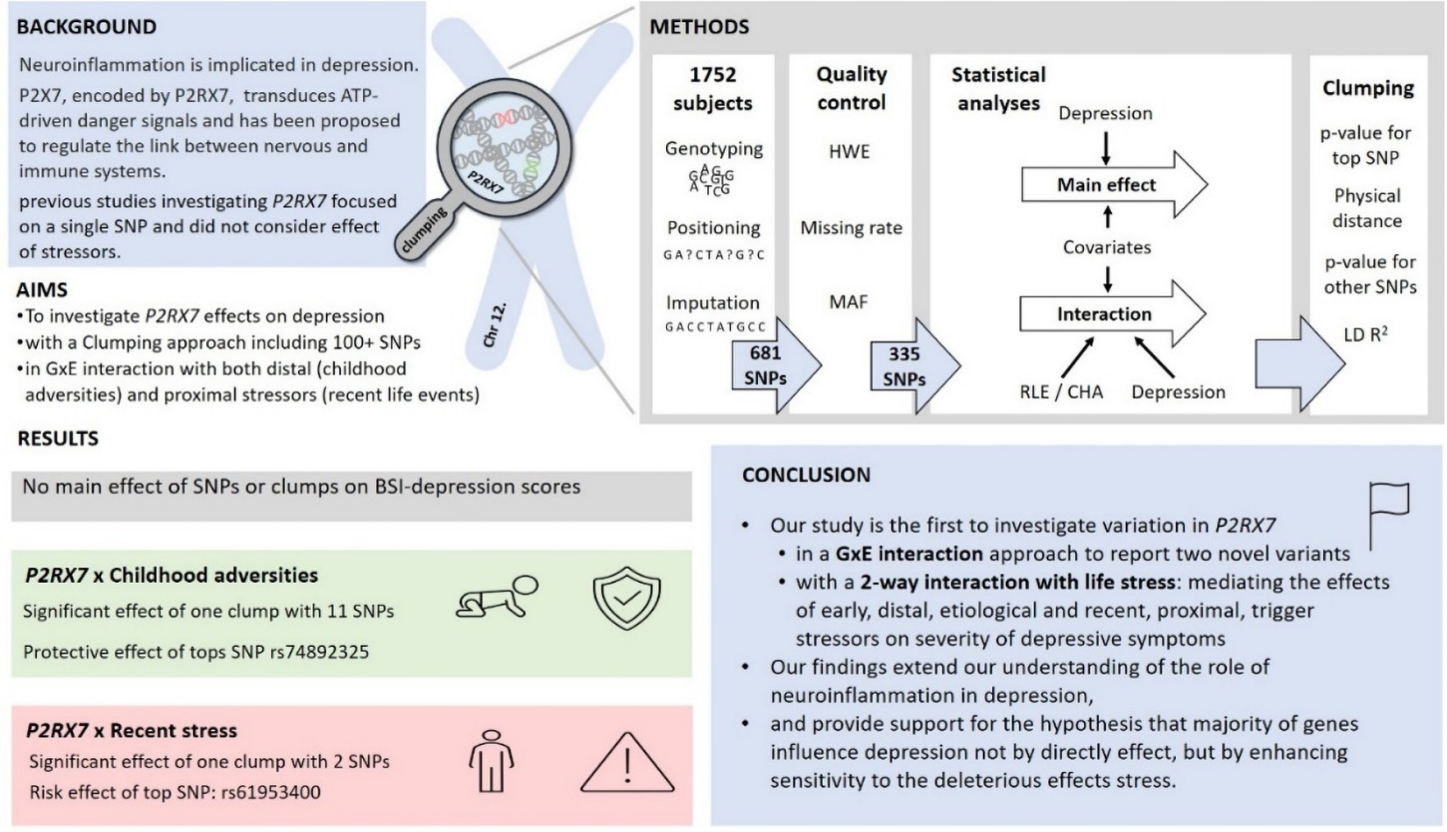
A summary of the study on the effect of *P2RX7* variation in interaction with childhood adversity and recent stress on the severity of depressive symptoms.

In our present study analysing variation along the *P2RX7* gene using a linkage disequilibrium-based clumping procedure, we identified two significant clumps. One clump, with top SNP rs74892325 significantly interacted with early childhood adverse experiences conveying a protective effect, and the other clump with top SNP rs61953400 significantly interacted with recent stressful life events conveying a risk effect. As no main effect for any investigated variants were observed, our results support that variation in *P2RX7* influences severity of current depressive symptoms in interaction with stress, mediating the effects of both early, distal stressors with an etiological effect and recent, proximal stressors, with a trigger effect. Our findings not only support previous results on the association of variation in this gene with depression, but emphasises its role in mediating the effects of stress (Fig 5).

Besides expression in peripheral immune cells, P2X7 receptors display a wide distribution across the brain located in microglia, central (and peripheral) neurons, and also show inconsistent and controversial expression in astrocytes and oligodendrocytes [40]. As a nonselective cation channel activated by sharp increases of extracellular ATP typically occurring during stressful conditions, P2X7 receptors execute ATP-driven danger signal transduction with relevant pathological and clinical implications [41]. The P2X7 receptor has been implicated as a central hub of divergent brain disorders including neurodegenerative disorders, neuropsychiatric disorders such as depression and schizophrenia, and brain tumours, due to its role in mediating several key processes [42,43]. While effects mediated by P2X7 receptor occur mainly through neuroinflammatory response and inflammasome activation [44], it also interacts and interferes with other stress and depression-related mechanisms [19], including impaired serotonin, noradrenaline, glutamate, GABA and NO release and transmission, and reduced neurogenesis and neuroplasticity [19,45–51]. In fact, P2RX7 is now being investigated in major depressive disorder as a potential novel therapeutic target [2,26] with several brain-penetrating antagonists in phase 2 and 3 clinical trials.

P2X7 receptors are activated by sharp increases of extracellular ATP release typically triggered by environmental stress resulting in NLRP3 inflammasome activation, which is maintained by a regenerative circuit even following termination of exposure, causing reduced BDNF levels, diminished synaptogenesis and neurogenesis, and damage to emotion and mood-regulation-relevant brain circuits [19]. P2RX7 is thought to play a role especially in chronic stress, where IL-1β driven microglial activation and neuroinflammation is upregulated. Efficacy of P2RX7 selective brain penetrant antagonists in animal models of chronic stress [52,53] also support a stress-mediated ATP-driven activation of the P2RX7 –NLRP3 –IL-Iβ pathway leading to proinflammatory microglial activation and neuroinflammation [2,41].

Human highly polymorphic *P2X7R* is located on 12q24.31 and has been reported to be associated with both unipolar major depression and bipolar disorder [20]. However, in spite of the increased interest in purinergic signalling and especially *P2RX7* in mood disorders, only a few studies focused on the role of variation in this gene. Most of these studies investigated one variant, rs2230912 of *P2RX7*, a nonsynonymous coding SNP causing an amino acid-exchange (Gln460Arg), associated with altered channel function enhancing Ca^2+^ influx, P2X7 dimerization, and further protein-protein interactions, thus influencing P2RX7-mediated signalling. Specifically, the G allele was associated with a gain-of-function phenotype for increased IL-1β release from monocytes in response to activation [24] suggesting that similar variation in *P2RX7* in microglia may similarly modify cytokine release, leading to neuroinflammation and changing the functional state of neural networks leading to increased vulnerability for mood disorders [23,25], although it is not yet understood how such SNPs increase the risk of mood disorders or, maybe even more importantly, how a loss of function would protect against them [2].

Following initial reports of an association between this variant and major depressive disorder [21], a few controversial replication attempts have been published with lack of a significant association in one meta-analysis in bipolar and unipolar depression [22], but a positive association with MDD in a more recent one including a greater number of studies [23]. In spite of the failure of some of the case-control studies in various types of affective disorders, dimensional approaches yielded confirmation for an association between rs2230912 and severity of depressive symptoms in bipolar disorder or combined unipolar and bipolar patient samples [54,55] or in bipolar samples [56] as well as with course characteristics of depression in unipolar and bipolar clinical samples including longer depressive episodes and more time spent ill [57]. In the above study of Vereczkei et al., not only rs2230912 but a minor-allele containing rs1718119-A ~ rs2230912-G ~ rs1653625-A haplotype was associated with a significantly higher depressive symptom score compared to the most frequent G~A~C haplotype among psychiatric patients with a major depressive episode, and also among diabetic patients, who are at higher risk for developing mood disorders.

The majority of studies investigating the role of *P2RX7* in depression have focused on rs2230912, but more recently it has been suggested that tagging all variations could help determine the extent of the role *P2RX7* plays in mood disorders [23], and understanding the role of other variations may shed further light on the association between *P2RX7* and disease susceptibility/protection [41]. Furthermore, given the role of P2X7 in mediating the effects of danger signals and stress, one possible reason for the above contradictory findings may be that pathological conditions as depression result from interaction between genes and the environment [23] yet studies failed to consider the interacting effects of life events.

The present study investigated the effects of several variants in *P2RX7* using a linkage disequilibrium-based clumping method, and focused on the interacting effects of stress with a complex approach, including distal and more etiological stressors in the form of early childhood adversities as well as proximal stressors playing a triggering role, in the form of recent negative life events occurring in the previous year. Furthermore, we also investigated severity of depressive symptoms using a dimensional approach. Several aspects of our findings are noteworthy. First of all, we identified two clumps with novel lead variants which have previously not been reported. None of the investigated variants have main effects on depression symptom score, that is, their effects became observable only in those exposed to moderate or severe stress in case of childhood adversities or severe stress in case of recent life events. This finding is in line not only with the supposed activation of *P2RX7* by stress and danger signals, but also with findings in our previous study, where using a Bayesian network analysis approach we reported that none of several variants previously associated with depression in seven genes including rs758311 in *P2RX7* showed relevance with respect to a complex depressive phenotype in the absence of stress, while the majority of them gained relevance in those exposed to moderate or severe recent life events [28]. Furthermore, our findings in detecting the effect of *P2RX7* variation on depression only in interaction with stress are in line with rodent models investigating humanized hP2Rx7 mice in a GxE scenario reporting that rs2230912 heterozygous mice exposed to chronic social defeat show higher anhedonia and anxiety compared to homozygotes [58], and concluding that *P2RX7* plays a role specifically in stress-related pathologies [26]. This conclusion is also supported by findings of enhanced *P2RX7* expression in the frontal cortex and hippocampus of mice exposed to chronic unpredictable stress or chronic restraint stress [59,60], while knockout mice studies suggest that absence of *P2RX7* confers both increased stress resilience and a phenotype with decreased depression-like characteristics [19]. Synthesising findings from several animal studies it has been hypothesised that *P2RX7* in interaction with stress predisposes to depression via profound effects on several processes interacting with environmental factors through the life span [26,58]. In summary, both pharmacological and genetic studies suggest that P2X7 receptor stimulation during stress leads to increased vulnerability and subsequent development of behavioural abnormalities associated with the neurobiology of depression [19,61,62]. Our finding showing the interaction of *P2RX7* variation with early and current stressors confirms these results.

The present study has several limitations. First, childhood adversity and recent life events were assessed retrospectively, and based on self-report of the subjects but not ascertained by other informants, which may contribute to distortion from several sources, including recall bias. Second, in a similar way, current depression severity was also based on a self-reported measure. Third, our population sample was relatively small, consisted in approximately two thirds of female subjects and limited to European white participants. Fourth, scoring childhood adversity and counting the number of recent negative life events does not take into consideration the differing severity of individual life events. Also, we considered objective occurrence of these life events and not their subjective meaning. Nevertheless, our study also has several strengths, including considering several hundred variants along the *P2RX7* gene with a clump method rather than just a single candidate SNP, employing a dimensional approach to capture current depression symptom severity, and using a GxE paradigm with two etiologically different types of stressors.

## Conclusions

In conclusion, our study is the first to investigate variation along the *P2RX7* gene in a large sample using a linkage disequilibrium-based clumping method and also a GxE interaction approach to report two clumps of variants with novel lead SNPs mediating the effects of distal and proximal stressors on the severity of depressive symptoms. Besides specifically focusing on the role of *P2RX7*, our findings extend our understanding of the role of neuroinflammation in depression, and also provide further support for the hypothesis that a great majority of genes involved in the background of depression do not act by directly leading to mood symptoms, but by enhancing sensitivity to the deleterious effects of different types of stressors.

## Supporting information

S1 Table *P2RX7* SNPs in the NewMood database in interaction with childhood adversities (CHA) with results in quality control steps and linear regression for BSI-depression. (XLSX)

S2 Table *P2RX7* SNPs in the NewMood database in interaction with recent life events (RLE) with results in quality control steps and linear regression for BSI-depression. (XLSX)
